# Supplementary material for: Tau hyperphosphorylation impairs cooperative binding to microtubules and perturbs organelle trafficking in neurons
Source: eLife. 2026 Sep 28;15:RP110011. doi: 10.7554/eLife.110011 (PMC13619133; doi:10.7554/eLife.110011)
Supplement: Peer review history

# Editor report: eLife Assessment

**DOI:** 10.7554/eLife.110011.3.sa0

**Authors:** Kassandra M Ori-McKenney

**Affiliations:** University of California, Davis United States

**Keywords:** Convincing, Solid, Important

In their **important** study, Beaudet, Berger and Hendricks provide a mechanistic link between disease-associated tau hyperphosphorylation, loss of cooperative tau envelope formation on microtubules, and dysregulation of axonal transport prior to aggregation. Using complementary in vitro reconstitution and human iPSC-derived neuronal assays with phosphodeficient and phosphomimetic tau constructs targeting 14 disease-relevant sites, the authors **convincingly** show that phosphorylation state alters tau organization on microtubules and differentially impacts kinesin- and lysosome-based transport. The evidence is **solid** and well aligned with the conclusions and will be of interest to the biophysical, cell biology and neurodegenerative communities.

# Referee report: Reviewer #1 (Public review)

**DOI:** 10.7554/eLife.110011.3.sa1

Summary:

This work by Beaudet and colleagues aims at exploring the effect of phosphorylation on the formation of tau envelopes and consequently on axonal transport both in vitro on reconstituted microtubules and in human excitatory neurons derived from IPSCs.

The authors found that a relatively widely used construct in which 14 serine or threonine residues often hyperphosphorylated in Alzheimer's disease are mutated to alanines (phosphodeficient) increases the density of tau envelopes compared to wildtype tau whereas a phosphomimetic (same residues mutated to glutamic acid) reduces envelopes density both in vitro and in human excitatory neurons derived from IPSCs.

By analysing the trafficking of different kinesins (KIF1a and KIF5C), they observed different effects of tau phosphorylation status on the movement of these two motors.

They then analyse transport of lysosomes by employing live imaging of lysotracker in human excitatory neurons derived from IPSCs transfected with wildtype, phosphodeficient or phosphomimetic tau observing that phosphodeficient tau seems to reduce transport of lysosomes while phosphomimetic increases transport compared to wildtype tau.

Strengths:

(1) The work aims to study a novel and underexplored topic in the tau field, tau envelopes, and investigate their relevance to Alzheimer's disease pathology.

(2) Experiments are well conducted and of high quality.

Weaknesses:

Relying only on in vitro reconstituted microtubules and human neurons derived from IPSCs leaves some doubts about the relevance of these results for Alzheimer's disease considering the embryonic state of IPSCs-derived neurons, but the authors clearly discuss this point.

# Referee report: Reviewer #2 (Public review)

**DOI:** 10.7554/eLife.110011.3.sa2

This manuscript examines how disease-associated hyperphosphorylation disrupts tau's role as a cooperative microtubule-binding regulator of intracellular transport. Using in vitro reconstitution assays and live-cell imaging in iPSC-derived neurons, the authors employ phosphomutant tau constructs (E14 to mimic hyperphosphorylation, AP to prevent phosphorylation) at 14 disease-associated residues to isolate phosphorylation effects independent of expression system-dependent PTM heterogeneity. The results show that hyperphosphorylated tau fails to form cooperative envelope-like structures on microtubules, instead binding diffusely and dissociating rapidly. In contrast, wild-type and phospho-resistant tau form cohesive envelopes that regulate motor protein access. At the single-molecule level, hyperphosphorylation reduces KIF5C inhibition while maintaining or enhancing KIF1A inhibition through altered processivity and detachment rates. In live neurons, hyperphosphorylated tau phenocopies tau knockout conditions, weakening tau-mediated inhibition of lysosome transport and increasing processive motility. The authors quantify tau binding using Gaussian mixture model-based image analysis and measure tau kinetics via FRAP, demonstrating that hyperphosphorylation-induced loss of cooperative binding correlates with dysregulated organelle transport. These findings establish a mechanism by which phosphorylation-driven disruption of tau's gatekeeper function on microtubules compromises axonal transport prior to aggregation in tauopathies.

Comments on revised version.

The authors did a good job responding to my comments and I support publication of the revised manuscript.

# Author comment: Author response

**DOI:** 10.7554/eLife.110011.3.sa3

**Authors:** Daniel Beaudet, Christopher L Berger, Adam G Hendricks

**Affiliations:** McGill University Montreal Canada; University of Vermont Burlington United States; Department of Bioengineering, McGill University Montreal Canada

The following is the authors’ response to the original reviews.

We thank the reviewers for their thoughtful and constructive feedback. In response, we substantially revised the manuscript and clarified the rationale for using in vitro reconstitution assays and iPSC-derived neurons to determine how tau hyperphosphorylation alters its interaction with microtubules and its regulation of intracellular transport. We have also more clearly articulated how these findings relate to neurodegeneration and discussed the limitations of the model systems used.

The primary concern of the reviewers was the justification for using COS7 cell lysates in reconstitution assays and iPSC-derived neurons as model systems. We have revised the manuscript to clarify that these experimental systems provided a means to isolate and examine how AD-related tau hyperphosphorylation alters tau-microtubule interactions and the regulation of intracellular transport. COS7 cells were selected because they are widely used for expression of mammalian proteins, including kinesins and tau. Human iPSC-derived neurons were chosen for their amenability to TIRF microscopy and transfection-based experiments, as well as the ability to compare CRISPR-generated tau knockout (MAPT-KO) neurons with their isogeneic control counterparts. Accordingly, we have revised the language throughout the manuscript to more clearly define the study’s objectives and emphasize that these systems were intentionally chosen as robust, well-controlled platforms for addressing specific mechanistic questions. We agree that they do not fully recapitulate AD pathology and that more representative models, such as mature, aged neurons or patient-derived neurons would be better suited to studying disease progression, and have included this limitation in the Discussion. However, because the central objective of this study was to dissect the mechanistic consequences of tau hyperphosphorylation on microtubule interactions and intracellular transport, we believe that these experimental approaches are well suited to address the questions asked.

We also more explicitly addressed how background levels of phosphorylation may contribute to the effects observed with the pseudo-phosphorylation model of AD-related tau perturbations. We’ve addressed this by citing recent studies (Fan et al., 2025; Siahaan et al., 2026; Moretto et al., 2026) that quantitatively assess phosphorylation across expression systems and clarified how our experimental design, which directly compares WT, AP and E14 tau, effectively minimize uncertainty arising from background phosphorylation. While some degree of background phosphorylation is likely to be present, any resulting effects would be expected to occur consistently across all tau phospho-variants. We now discuss the limitation of our study that we did not directly quantify phosphorylation levels in cells.

The reviewers also expressed concern about the potential influence of endogenous microtubule-associated proteins present in lysates and differences in tau occupancy on microtubules contributing to motility outcomes. To address this, we included additional analyses correlating tau intensity along microtubules with kinesin motility. We also expanded the Discussion to consider how tau competes with other MAPs for microtubule binding and how phosphorylation-dependent changes in tau–microtubule interactions may alter the MAP landscape. Consequently, the transport phenotypes observed with different tau phospho-variants may reflect both direct effects of tau and indirect effects arising from changes in MAP occupancy and competition on the microtubule lattice.

We provide detailed, point-by-point responses to each reviewer comment below. We appreciate the thoughtful feedback from reviewers and are confident that the revisions, which include clearer language, strengthened justification of the experimental approaches, and additional supporting analyses, have substantially improved the clarity, rationale, and overall impact of the study.

**Public Reviews:**

**Reviewer #1 (Public review):**

Summary:

This work by Beaudet and colleagues aims at exploring the effect of phosphorylation on the formation of tau envelopes and consequently on axonal transport, both in vitro on reconstituted microtubules and in human excitatory neurons derived from IPSCs.

The authors found that a relatively widely used construct in which 14 serine or threonine residues, often hyperphosphorylated in Alzheimer's disease, are mutated to alanines (phosphodeficient), increases the density of tau envelopes compared to wildtype tau, whereas a phosphomimetic (same residues mutated to glutamic acid) reduces envelope density both in vitro and in human excitatory neurons derived from IPSCs.

By analysing the trafficking of different kinesins (KIF1a and KIF5C), they observed different effects of tau phosphorylation status on the movement of these two motors.

They then analyse transport of lysosomes by employing live imaging of lysotracker in human excitatory neurons derived from IPSCs transfected with wildtype, phosphodeficient or phosphomimetic tau, observing that phosphodeficient tau seems to reduce transport of lysosomes while phosphomimetic increases transport compared to wildtype tau.

Strengths:

(1) The work aims to study a novel and underexplored topic in the tau field, tau envelopes, and investigate their relevance to Alzheimer's disease pathology.

(2) Experiments are well conducted and of high quality.

Weaknesses:

Relying only on in vitro reconstituted microtubules and human neurons derived from IPSCs leaves some doubts about the relevance of these results for Alzheimer's disease, considering the embryonic state of IPSCs-derived neurons.

We agree with the reviewer that iPSC-derived neurons represent an immature state compared with the neurons most affected in Alzheimer’s disease. However, iPSC-derived neurons and in vitro reconstitution are robust experimental approaches that provide insight into (1) the effects of hyperphosphorylation on tau’s cooperative microtubules association and envelope formation, (2) how tau hyperphosphorylation affects the motility of kinesin motors that are sensitive to regulation by tau, and (3) how tau hyperphosphorylation alters the bi-directional transport of endogenous degradative organelles such as lysosomes. Our studies reveal the molecular effects of how hyperphosphorylation influences tau’s role in regulating intracellular transport and we believe that these findings will help to inform future studies examining how tau-related dysfunction first influences axonal transport, which would be expected to alter axonal health and homeostasis prior to the more severe pathological effects observed at later disease stages.

We have included a paragraph under the subheading ‘Limitations of this study’ in the Discussion section to better contextualize our findings within the broader effort to understand tauopathies and Alzheimer’s disease. We clarify the limitations of using in vitro reconstitution and iPSC model systems on pages 20 and 21.

**Reviewer #2 (Public review):**

This manuscript examines how disease-associated hyperphosphorylation disrupts tau's role as a cooperative microtubule-binding regulator of intracellular transport. Using in vitro reconstitution assays and live-cell imaging in iPSC-derived neurons, the authors employ phosphomutant tau constructs (E14 to mimic hyperphosphorylation, AP to prevent phosphorylation) at 14 disease-associated residues to isolate phosphorylation effects independent of expression system-dependent PTM heterogeneity. The results show that hyperphosphorylated tau fails to form cooperative envelope-like structures on microtubules, instead binding diffusely and dissociating rapidly. In contrast, wild-type and phospho-resistant tau form cohesive envelopes that regulate motor protein access. At the single-molecule level, hyperphosphorylation reduces KIF5C inhibition while maintaining or enhancing KIF1A inhibition through altered processivity and detachment rates. In live neurons, hyperphosphorylated tau phenocopies tau knockout conditions, weakening tau-mediated inhibition of lysosome transport and increasing processive motility. The authors quantify tau binding using Gaussian mixture model-based image analysis and measure tau kinetics via FRAP, demonstrating that hyperphosphorylation-induced loss of cooperative binding correlates with dysregulated organelle transport. These findings establish a mechanism by which phosphorylation-driven disruption of tau's gatekeeper function on microtubules compromises axonal transport prior to aggregation in tauopathies. The paper provides interesting new knowledge for the field, but there are outstanding concerns that could be further addressed by the authors to strengthen and clarify the current manuscript:

(1) Lack of Phosphatase-Treated Control and Explicit WT Phosphorylation Quantification

Wild-type tau expressed in insect and mammalian cells is known to be phosphorylated by endogenous kinases (eg, GSK3, CDK5, MARK). The manuscript acknowledges this in the Discussion but provides no phosphatase-treated lysate control or quantification of endogenous phosphorylation on WT tau via phospho-specific Western blots. This leaves ambiguity about whether observed differences between WT and E14 reflect purely the introduced mutations or confounding baseline differences in phosphostate content.

Tau contains ~85 putative phosphorylation sites and is modified by several kinases in cells. Studies by Siahaan et al. (2026) and Fan et al. (2025) provide detailed insight into tau phosphorylation heterogeneity, its role in protecting the microtubule lattice from severing enzymes, and the implications of phosphorylation patterns for aggregate formation. We reference these papers and include detailed description of these findings when initially establishing our justification for using pseudo-phosphorylation model.

We used a pseudo-phosphorylation approach to test the effects of phosphorylation of specific residues in the proline-rich region and the pseudo-repeat domain in the C-terminus, which together with the microtubule-binding repeats, establish the minimal regions required for tau’s cooperative microtubule binding (Tan et al., 2019). This system enabled us to dissect the effects of tau phosphorylation without the added complexities of heterogeneity and multiple isoforms of tau that would otherwise be endogenously expressed. We’ve clarified these points in the revised manuscript (Pages 6, 7, 17, and 18).

Background phosphorylation in the different phospho-variants used might contribute to the observed changes in tau’s MT interactions and regulation of transport. However, based on our results and the significance in the changes between the different phospho-variants, even if there is some basal level of phosphorylation, the results indicate that the effects of the pseudo-phosphorylation sites are strong enough to make observable changes above the basal levels of phosphorylation (see p. 6 of the revised manuscript).

Disease-associated phosphorylation is likely more heterogeneous and dynamic than the pseudo-phosphorylation mutants used here, and phosphorylation at different sites may differentially regulate tau function (see p. 21 of the revised manuscript).

(2) Limited Normalization of Motor Effects to Measured Tau Lattice Occupancy

Although kinesin trajectories are classified inside vs. outside tau envelopes (inherently normalizing to local tau density), motor parameters are not systematically reported as functions of tau fluorescence intensity across all constructs. Co-purifying MAPs or microtubule-modifying enzymes in cell lysates is not quantified or excluded, leaving residual uncertainty about tau-specificity of observed motor inhibition. This should be at least acknowledged in the results section.

As noted by the reviewer, it is challenging to compare conditions where the occupancy of tau on microtubules is dissimilar across conditions. To address this point, we performed a Spearman’s correlation analysis to compare how tau intensity affects kinesin dynamics along microtubules (Fig S3G). On page 12, our results show that kinesin dynamics are generally reduced in regions of high tau occupancy. However, in regions of comparable higher intensities, KIF5C is less inhibited by E14 tau, whereas KIF1A is less inhibited by AP tau.

On pages 12 and 13, we acknowledge that while effects from other MAPs or motor proteins could potentially affect kinesin motility, we would expect that any effect from residual lysate components would be similar across tau phospho-variants.

(3) Insufficient Citation of Prior Neuronal Tau Envelope Evidence

In the Introduction, the authors state, "it was an open question if tau forms envelopes in neurons," but this understates existing evidence. Tan et al. (2019) report tau neuronal staining consistent with envelope formation, while Siahaan et al. (2021) provide more direct evidence in non-neuronal cells. The framing should acknowledge and integrate these prior findings.

We agree with the reviewer that evidence from several studies using reconstitution systems, fixed neurons, and live cultured cells provides evidence of tau envelope formation in neurons. Specifically, tau envelopes have been observed along taxol-stabilized or GMPCPP-capped GDP microtubules in vitro (e.g., Dixit et al., 2008; Monroy et al., 2018; Tan et al., 2019; Siahaan et al., 2019), in 4% PFA-fixed and Triton X-100–extracted DIV7 mouse hippocampal neurons (Tan et al., 2019), and in live, non-neuronal U-2 OS cells following taxol treatment (Siahaan et al., 2022) or elevated pH (Siahaan et al., 2024). To our knowledge, our study is the first to demonstrate tau envelope formation in live neuronal cells under normal cell culture conditions. We revised the introduction (see pages 3 and 4) to more precisely position our findings within the context of prior studies.

(4) Unclear Wording on Expression System-Dependent Phosphorylation

The sentence "The phosphostate of tau is strongly dependent on the expression system" requires rewording. It is ambiguous whether this refers to the final phosphostate achieved after expression or the inherent phosphorylating capacity of each system. Clearer language would strengthen the methodological justification.

On pages 6 and 7, we clarify the rationale for using COS7 cells to express GFP-tau and elaborate on recent papers demonstrating how different expression systems used to study tau (e.g., bacterial, insect, mammalian) produce tau with variable phosphorylation patterns (Siahaan et al., 2026; Fan et al., 2025).

(5) Insufficient Quantification of Motor and Lysosome Transport Effect Magnitudes in Results Section

The data on molecular motor motility and lysosome transport are densely described. The magnitude of effects (fold-changes, percentage differences) should be explicitly stated in the Results section when first presenting findings to orient readers to biological significance. For example, effect magnitudes for lysosome run lengths, velocities, and directional bias should be quantified in text, not left to figure inspection.

We now incorporate the relevant quantifications in the text.

(6) Incomplete Discussion of Projection Domain Necessity for Envelope Formation

The Discussion states the projection domain is "a critical regulator of both tau-tau and tau-microtubule interactions," but does not engage with prior domain dissection work. Tan et al. (2019) found that the entire projection domain is not necessary for envelope formation in vitro. The authors should discuss which projection domain regions are specifically regulated by phosphorylation vs. required for cooperativity, providing a more nuanced interpretation than implied by their current framing.

Tan et al. (2019) demonstrated that part of the proline-rich region (residues 198–244) within the N-terminal projection domain and the pseudo-repeat region within the C-terminus, together with the microtubule-binding repeats are the minimal region required to maintain tau’s ability to form cooperative envelopes along microtubules. We revised the text to better incorporate this previous work into the discussion and place our findings within this context. Our work demonstrates how phosphorylation within the proline-rich region and pseudo-repeats are important regulators of tau–tau cooperativity.

**Recommendations for the authors:**

**Reviewer #1 (Recommendations for the authors):**

(1) It is unclear how the method implemented by the authors to identify tau envelopes works exactly (GMM and BIC) and how appropriate it is. It does not appear similar to what others have done in the literature on tau envelopes. Moreover, when checking the intensity plots along microtubules, both in Figures 1C and 2A, one is left to wonder if the observed differences are not simply caused by the different thresholds. Indeed, the intensity profiles do not seem greatly different between conditions in Figure 1C, whereas it is evident that the threshold is quite different, with it being lower for AP tau and higher for E14 tau, which explains the differences in how many envelopes are detected. The authors could also try to quantify in a different way (e.g. even just a threshold based on Average + SD) to see if the results remain the same?

We used a GMM/BIC approach to avoid biased comparisons of tau envelope formation on microtubules across phospho-conditions. In TIRF assays, intensity signals are inherently inconsistent, making it difficult to directly compare fluorescence intensity signals on different microtubules across regions within the same field of view. Additionally, tau distribution between microtubules and in solution varied between conditions (e.g., background tau signal is elevated in E14 conditions compared to WT or AP tau). Given these challenges, we quantified and compared tau intensities on a per-microtubule basis, which produced more robust results. While we initially attempted the reviewer’s suggested approach of using average + SD, per-microtubule variability in minimum and maximum signal, along with differences in local background, prevented the ability to set a threshold that reliably captured intensity differences along microtubules across and within replicates. We now more clearly explain why we chose this approach (see page 7).

(2) The authors should discuss the possibility that the presence of a GFP tag at the N terminus of tau could affect the formation of envelopes, given the importance of this region. Also, they refer to tau GFP in some points of the text and other times to GFP tau. As it would seem they have always used tau tagged at the N terminus, they should refer to GFP-tau in order to avoid confusion in the position of the tag.

We agree with the reviewer that the position of the N-terminal GFP could influence the projection domain, However, all tau constructs carry the GFP tag at the same position and differ only in their phospho-site mutations. The correct nomenclature for “GFP-tau” is now consistently used throughout.

(3) Previous work (Tan et al., 2019) has shown that different isoforms of tau have different propensities to form tau envelopes. The authors should specify in each figure which isoform of tau they are expressing.

The tau isoform used throughout this study is 4R0N. The tau isoform is clearly identified in the revised text.

(4) Figure 1 C-E: It would be interesting to see the size of envelopes quantified, also.

We now include a comparison of the mean envelope width for each phospho-variant (Fig 1F).

(5) Figure 2F: As the FRAP experiment is not on tau envelopes but generally on axonal tau, this needs to be clearly stated to highlight how this limits the link between the different FRAP dynamics and the behaviour of tau envelopes.

We changed the text to indicate that we perform FRAP on axonal tau and not specifically tau envelopes.

(6) The authors should discuss whether they expect Kif1a and Kif5c to be responsible for transporting lysotracker-positive vesicles in neurons? This does not seem to be the case based on a quick literature search. If these are not the motors responsible for the transport of lysosomes, why do the authors decide to look at the transport of these organelles and not others? Also, what is the rationale for studying the transport of lysosomes, an organelle that is mainly transported retrogradely, after identifying defects in kinesin transport? The authors could either study in vitro the effect of tau phosphorylation on the movement of a kinesin more directly linked to lysosomes (e.g. KIF5B, KIF1B) or study the transport of some other organelle which is mediated by KIF1A and KIF5C.

We revised the text to clarify this point. Several studies have shown that kinesin-1 and -3 are strongly inhibited by tau, whereas kinesin-2 and dynein are less sensitive (Hoeprich et al., 2017; Chaudhary et al., 2018; and others). Within this context, we asked how phosphorylation alters tau’s inhibitory effects on motors that are most sensitive to tau. The in vitro reconstitution assays were not intended to isolate the effects of tau on lysosome-specific motors. Rather, they were used to determine how tau phosphorylation affects representative kinesin-1 and -3 motors that drive a substantial fraction of anterograde axonal transport and are among the most sensitive to tau-mediated regulation.

We next examined lysosome transport using LysoTracker to investigate how tau phosphorylation influences bidirectional cargo transport. Lysosomes are transported by teams of kinesin-1, -2, -3, and dynein, making them a useful model for assessing the consequences of tau regulation in a more physiological context. Current models of bidirectional transport proposed that cargo movement emerges from tug-of-war, which is a result of a balance of forces generated by opposing motors. Under this assumption, strong inhibition of kinesin by tau would be expected to reduce anterograde transport and/or enhance retrograde transport by shifting this balance towards dynein. We have clarified throughout the manuscript that our goal was to determine how tau phosphorylation affects bidirectional transport and to interpret these findings within this context. Because defects in degradative pathways are thought to contribute to neurodegeneration, these experiments may also provide insight into how tau hyperphosphorylation disrupts lysosome function during disease.

Although KIF5C and KIF1A are not the primary kinesin homologs responsible for lysosome transport, we expect that other kinesin-1 and kinesin-3 motors respond similarly to tau. The in vitro findings provide mechanistic insight into how tau phosphorylation could alter motor function and ultimately contribute to changes in lysosome trafficking and distribution within axons. On page 19, we further clarify that the magnitude of tau-mediated regulation is likely to vary among kinesin family members due to differences in their intrinsic motor properties, and that the effects on lysosome transport are therefore expected to be more nuanced than those observed for individual motors in vitro.

(7) In the trafficking experiments with lysotracker in human excitatory neurons, there seems to be a large fraction of anterogradely transported lysotracker-positive organelles. Based on a quick search, it would appear this occurs frequently in IPSC-derived neurons, but it's not the case in primary neurons (see, for example, Kulkarni et al., 2022). Given that IPSCs-derived neurons maintain an immature embryonic maturation status (as correctly stated by the authors when mentioning that they express mainly 3R tau) and that neuronal maturation influences transport in primary neurons (e.g. Moutaux et al., 2018), the authors should discuss these aspects highlighting the possible limitations, especially considering the claim of importance of their results for Alzheimer's disease, a pathology that hits neurons at full maturation stages. Alternatively, they could perform a similar experiment in murine neurons at mature stages.

See response to comments from reviewer 1 under ‘weaknesses’.

(8) In the context of the previous point, the immature phenotype of IPSCs could explain the apparent discrepancy between the results obtained by these authors and previously published work (Hallinan et al., 2019), which found that mature hippocampal neurons expressing E14 tau had reduced transport of lysosomes. Moreover, these authors also described patches of higher intensity of tau along the axons formed by E14 tau compared to WT tau, which are closely reminiscent of tau envelopes. The authors should discuss these discrepancies.

We agree that there are discrepancies between our findings and those reported by Hallinan et al. (2019). In that study, E14 tau was shown to misfold in cultured mouse hippocampal neurons, forming MC1-positive axonal aggregates that impair lysosome transport. In contrast, we observe nearly the opposite effect: E14 tau remains diffusely distributed in axons and produces a phenotype resembling tau knockout conditions, with enhanced lysosome transport.

These differences may stem from methodological factors, including the neuronal models used (murine hippocampal cultures versus human iPSC-derived neurons), fixation and immunolabeling compared with live-cell imaging, differences in neuronal maturity (DIV), and the presence of endogenous tau versus our knockout-and-rescue approach. Importantly, tau aggregation may reflect later stages of disease progression, where aggregates physically clog axons leading to obstructed axonal transport rather than tau acting as a regulatory “roadblock” to specific motor proteins. We now cite this paper and compare our results with this study and discuss these discrepancies and their potential implications in the ‘Limitations of this study’.

(9) Figures 4 and 5 are quite hard to read. Perhaps the distinction between proximal, mid and distal axon, although valuable, could be moved to the supplementary, maintaining an overall average, or the most significant of the 3 in the main figures to improve readability?

We made substantial revisions to figures 4 and 5 and the associated analysis. Because the effects of tau on lysosomal transport were largely consistent across proximal, mid, and distal axonal regions, we combined these datasets and report overall transport trends within the axon (from ~50 µm distal to the AIS to ~50 µm proximal to the growth cone). The region-specific analyses and figures showing lysosome motility in each axonal segment have been moved to Supplementary Figure S4.

(10) In the discussion, the authors write an entire paragraph on how their results are important to stress the importance of the N terminus of tau in the formation of tau envelopes. This is based on the fact that most of the residues mutated in the phosphomimetic and phosphodeficient constructs are located in the N-terminal projection domain. However, some of these residues are located in the C terminus of tau, which also appears to have a role in tau envelope formation (Tan et al., 2019). The experiments presented do not discriminate the phosphorylation of which of the 14 residues is important to mediate the effects. Hence, I feel this paragraph needs to be toned down or removed entirely.

See response to comment 6 from reviewer 1.

(11) The authors make a point of using tau produced in mammalian cells in the experiments performed in vitro, stressing the advancement compared to previous work that used tau produced in bacteria or insect cells. Although this is certainly closer to physiological conditions, the production is done in cancerous kidney cells, so I feel the author should highlight that neurons might drive a distinct phosphorylation pattern. Could recombinant tau be produced in neuroblastoma cells?

In the revised manuscript, we’ve addressed this comment in the “Limitations of this study” page 21. We used COS-7 cells, which are not cancerous but immortalized fibroblast-like cells derived from African green monkey kidney obtained from ATCC. These cells were chosen because of their widespread use for protein expression and their high transfection efficiency. We agree that the physiology of COS-7 lysates is not directly comparable to that of neurons. Our intention was to convey that proteins expressed in mammalian systems undergo post-translational modifications and are produced by cellular machinery that more closely resembles neuronal systems than bacterial or insect expression platforms. Although neuronal cell lines such as neuroblastoma cells may appear more physiologically relevant, they are often difficult to transfect (Alabdullah et al., 2019). This can create practical challenges in equalizing protein concentrations and obtaining sufficient amounts of overexpressed tau from lysates. While methods exist to improve transfection efficiency, there is no literature that we found stating that these cells would yield protein expression characteristics more comparable to neurons than COS-7 cells. A more comprehensive evaluation of alternative expression systems would require a substantially deeper literature search or systematic characterization of multiple cell lines, which falls beyond the scope of this manuscript. Therefore, we relied on the robust COS-7 expression system and will clarify this rationale in the revised manuscript.

Alabdullah AA, Al-Abdulaziz B, Alsalem H, et al. Estimating transfection efficiency in differentiated and undifferentiated neural cells. BMC Res Notes. 2019;12(1):225
